# Lessons and Implications from Genome-Wide Association Studies (GWAS) Findings of Blood Cell Phenotypes

**DOI:** 10.3390/genes5010051

**Published:** 2014-01-28

**Authors:** Nathalie Chami, Guillaume Lettre

**Affiliations:** Montreal Heart Institute, Faculté de Médecine, Université de Montréal, 5000 Bélanger Street, Montréal, QC H1T 1C8, Canada; E-Mail: nathalie.chami@mhi-humangenetics.org

**Keywords:** GWAS, hemoglobin, hematocrit, red blood cell, erythrocyte, white blood cell, leukocyte, platelet, human genetics

## Abstract

Genome-wide association studies (GWAS) have identified reproducible genetic associations with hundreds of human diseases and traits. The vast majority of these associated single nucleotide polymorphisms (SNPs) are non-coding, highlighting the challenge in moving from genetic findings to mechanistic and functional insights. Nevertheless, large-scale (epi)genomic studies and bioinformatic analyses strongly suggest that GWAS hits are not randomly distributed in the genome but rather pinpoint specific biological pathways important for disease development or phenotypic variation. In this review, we focus on GWAS discoveries for the three main blood cell types: red blood cells, white blood cells and platelets. We summarize the knowledge gained from GWAS of these phenotypes and discuss their possible clinical implications for common (e.g., anemia) and rare (e.g., myeloproliferative neoplasms) human blood-related diseases. Finally, we argue that blood phenotypes are ideal to study the genetics of complex human traits because they are fully amenable to experimental testing.

## 1. Genetics of Red Blood Cells, White Blood Cells and Platelets

Blood is mostly composed of plasma and blood cells and plays a major role in a variety of functions involved in general human homeostasis: it transports oxygen, nutrients and hormones to tissues, removes waste, performs immunological functions and contributes tissue damage repair through coagulation. The main three blood cell types carry out most of these activities: red blood cells (RBC, or erythrocytes) transport oxygen, white blood cells (WBC, or leukocytes) coordinate some of the immune responses, and platelets are the bricks that form blood clots to prevent excessive bleeding. All of these cell types originate through proliferation and differentiation from common precursors (hematopoietic stem cells) [1].

An aberrant number, size or feature of the three main blood cell types characterizes multiple human diseases (Table 1). In many cases, the triggering factor is of environmental origin, often poor nutrition or infections (e.g., malaria, HIV). Germline and somatic mutations can also cause severe blood disorders, such as mutations in glucose-6 phosphate dehydrogenase (*G6PD*) which is responsible for chronic hemolytic anemia or mutations in oncogenes or tumor suppressor genes that result in leukemia. It is also known that blood cell phenotypes vary between healthy individuals, and that some of this inter-individual variation is controlled by genetics. In a large study of healthy Sardinians (*N* = 6,148), the heritability estimates for RBC, WBC and platelet counts were, respectively, 0.67, 0.38 and 0.53 [2]. Similar heritability estimates were obtained when analyzing phenotype concordance in healthy monozygotic and dizygotic twins from the United Kingdom [3]. These results indicate that a large fraction of the phenotypic variation in these blood traits is controlled by DNA sequence variants segregating in healthy individuals.

**Table 1:** Main blood cell traits routinely measured in standard complete blood count (CBC).

Trait	Description	Unit
Red blood cell (RBC) count	Count of RBC per microliter	Million cells per microliter (×10^6^/µL)
Hemoglobin (HGB)	Hemoglobin concentration	Gram per deciliter (g/dL)
Hematocrit (HCT)	Fraction of blood that contains hemoglobin	Percentage (%)
Mean corpuscular hemoglobin (MCH)	Amount of hemoglobin per RBC	Picogram (pg)
Mean corpuscular volume (MCV)	Average volume of RBC	Femtoliter (fL)
MCH concentration (MCHC)	Hemoglobin divided by hematocrit	Gram per deciliter (g/dL)
RBC distribution width (RDW)	Distribution of RBC volume	Percentage (%)
White blood cell (WBC) count	Number of WBC per liter (include all main subtypes)	Billion cells per liter (×10^9^/L)
Platelet (PLT) count	Number of PLT per liter	Billion cells per liter (×10^9^/L)
Mean platelet volume (MPV)	Average platelet volume	Femtoliter (fL)

The clinical importance of this heritable variation in blood cell phenotypes is unclear. However, it is interesting that epidemiological studies have detected links between WBC or platelet counts and the risk to suffer from cardio- and cerebrovascular diseases [4,5,6]. As for most epidemiological observations, however, it is difficult to determine if changes in hematological parameters are pathological or reflect consequences of disease manifestation. Using Mendelian randomization methodologies, in which inherited genetic variants associated with hematological traits are used as instruments to test the causal effect of the traits on diseases, may provide an answer to this question [7]. Such an approach was successfully used to determine that LDL-cholesterol and triglyceride levels, but unlikely HDL-cholesterol levels, are causes of coronary artery diseases [8,9]. Understanding how DNA polymorphisms modulate blood cell phenotypes in health (and diseases) could provide new opportunities to study hematopoiesis, improve their use in medicine as biomarkers and maybe even help in the development of new drugs. To this list, we would also add that hematological traits are ideal phenotypes to further our understanding of the genetics of human complex diseases and traits because experimental systems exist to functionally validate genetic findings.

## 2. Genome-Wide Association Studies (GWAS) for Blood Cell Phenotypes

Before GWAS, little was known about the role of SNPs and other common DNA sequence variants on normal variation in blood cell phenotypes. Candidate gene DNA sequencing experiments have identified mutations in the globin loci, but also in the erythropoietin receptor (*EPOR*) and hemochromatosis (*HFE*) genes [10,11]. Genome-wide linkage studies also found a few reproducible signals, most notably a linkage peak on chromosome 6q23 that encompasses the MYB transcription factor [12,13]. These findings could not, however, explain the heritability of these blood cell phenotypes in normal individuals.

**Figure 1:**
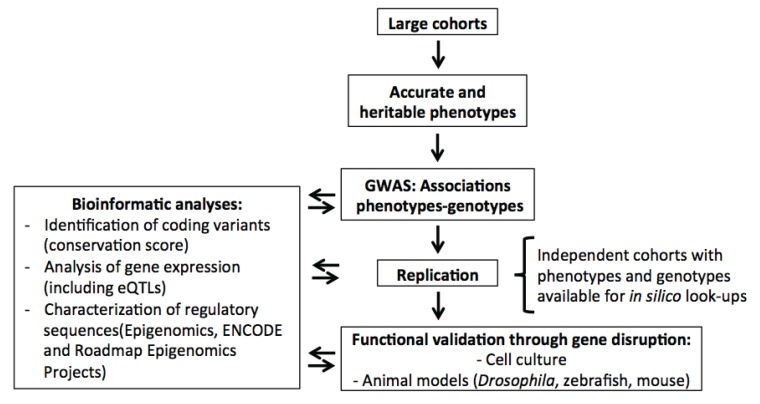
Ideal study design to identify single nucleotide polymorphisms (SNPs) associated with human complex traits and diseases using genome-wide association studies (GWAS). For blood cell phenotypes, GWAS were particularly successful because sample sizes are large, phenotypes are easy to measure and are accurate, and well-characterized experimental models already exist.

As for many other complex human traits and diseases, the capacity to test associations with genotypes across the genome by GWAS opened a new world. Prior to the GWAS era, genetic association studies often had sample sizes that were too small and were limited to testing only known genes [14]. With GWAS, it became possible to genotype all genes independently of previous knowledge. Blood cell traits are particularly amenable to the GWAS approach because they are routinely and accurately measured in large cohorts, and initial findings can be tested for replication in other cohorts because it is easy to harmonize these phenotypes (Figure 1) [15]. In general, one of the main challenges for GWAS has been to pinpoint functional genes and variants associated with a given trait. Although this remains a challenge, blood cell traits are particularly well-suited for genetic and functional follow-up. As mentioned earlier, fine-mapping by dense genotyping and DNA re-sequencing is possible because the traits are usually available in most cohorts or biobanks, including participants of different ethnicities (see below). There is also the possibility to test the functions of new genes in cell culture systems or model organisms because the phenotypes are often cell autonomous and the assays already well-developed. Using this approach, investigators showed that SNPs at 6p21.1 modulate erythrocyte traits through a regulatory effect on the cyclin D3 (*CCND3*) gene [16]. Large-scale gene silencing and other functional experiments in fruit flies, zebrafish and mice were also used to validate several new genes involved in platelet and RBC development within loci identified by GWAS [17,18].

All the steps described in Figure 1 now take advantage of powerful bioinformatic tools and other resources freely available on the web. For instance, comparative genomics has identified DNA bases that are conserved through evolution and therefore more likely to be functionally important [19]. There are also software that can predict based on conservation and physicochemical properties whether a DNA polymorphism that changes an amino acid is likely detrimental or not [20,21]. We can also quickly query large gene expression datasets to determine if the genes near an associated SNP are expressed in the relevant tissue(s) for the phenotypes of interest (as an example, see reference [22]). And when genotypes are available, it is possible to test *in silico* if the GWAS SNPs (or SNPs in linkage disequilibrium) control gene expression through regulatory mechanisms; that is, if the variants are expression quantitative trait loci (eQTL) [23]. The ENCODE and Roadmap Epigenomics Projects have used next-generation DNA sequencing applications, including DNAse I hypersensitive sites mapping and chromatin immunoprecipitation with antibodies against several histone tail modifications (ChIP-seq), to define regulatory sequences in human cell lines and tissues [24,25,26]. Using a complementary approach (FAIRE-seq), Paul *et al*. identified regions of open chromatin in primary human blood cells and showed that SNPs associated with RBC and platelet phenotypes are enriched in these regions [27]. All this vast genomic information is useful in prioritizing causal genes and variants at GWAS loci, and investigators are developing algorithms to facilitate its integration [28,29].

Several GWAS for hematological traits have already been published [17,18,30,31,32,33,34,35,36,37,38,39,40,41,42,43,44,45,46]. The largest studies, carried out in Europeans or individuals of European ancestry, have so far identified at genome-wide significance (*p*-value < 5 × 10^−8^) 75, 10 and 68 SNPs associated with RBC, WBC and platelet traits respectively [17,18,45]. The lower number of SNPs associated with WBC count could be explained by a lower heritability (see above), but also because the sample size for the WBC GWAS was smaller (*N* = 11,823) in comparison with the GWAS for RBC (*N* = 135,367) and platelet (*N* = 66,867) traits. Despite their large number, these variants only explain a small fraction of the heritable variation in these phenotypes (<10%). They are, however, not random but clustered near genes involved in relevant biological pathways and enriched for regulatory functions by expression quantitative trait loci (eQTL) and epigenomic analyses. Most loci are associated with a single blood cell type but by comparing the different studies, we found seven loci that are associated with at least two different cell types (Table 2). These include *SH2B3*, a gene that encodes the adapter protein LNK that interacts with JAK2 and modulates JAK-STAT signaling in hematopoietic cells, and *MYB*, that encodes a transcription factor essential for definitive hematopoiesis. Both *SH2B3* and *MYB* SNPs are associated with the three main blood cell types. The other loci presented in Table 2 include genes associated with a combination of two phenotypes, maybe suggesting different functions in different hematopoietic lineages.

**Table 2:** Loci identified by GWAS that carry SNPs associated with at least two of the three main blood cell types. For each association, we report the ethnic group in which the genetic associations were found. We also listed only one gene per locus, although for many loci, the causal gene is unknown. RBC: red blood cell; WBC: white blood cell.

Locus	Location	RBC	WBC	Platelet	References
*TMCC2*	1q32.1	Caucasian		Caucasian	[17,18]
*ARHGEF3*	3p14.3	African American		Caucasian	[17,30,36,38]
*LRRC16A*	6p22.2	African American		African American	[31,37]
*HBS1L-MYB*	6q22-q23.3	African American/Caucasian/Japanese	Caucasian	African American/Caucasian	[17,18,31,32,34,35,37]
*IL-6*	7p21		Japanese	Japanese	[47]
*RCL1*	9p24.1-p23	Caucasian		Caucasian/Japanese	[17,18,32,34]
*SH2B3*	12q24	Caucasian	Caucasian	Caucasian/Japanese	[17,32,33,34,35,38]

### Some Loci Associated with Blood Cell Traits Are Population-Specific

It is difficult to compare association results for hematological traits across different populations because the sample size of the respective GWAS, and thus the statistical power to discover associations, is very different. For instance for RBC phenotypes, the largest studies in Caucasians and African Americans included, respectively, 135,367 and 16,496 participants [18,31]. Despite this caveat, many of the loci found in African Americans or Asians were also present in Caucasians; this general transferability of results across ethnic groups has been observed for other complex human traits [48,49]. For blood cell traits, however, there are notable exceptions. A SNP upstream of the Duffy antigen/receptor for chemokines (*DARC*) gene explains a large fraction of the variation in WBC and neutrophil counts, and is responsible for benign neutropenia [50]. This variant, which is monomorphic in Caucasians, is under positive selection in persons of African ancestry because it provides protection against *Plasmodium vivax* malaria infections. Similarly, genetic variation near the *α-globin*, the *β-globin* and the *G6PD* genes are associated with RBC indices in Africa-derived populations and are relatively common in frequency because they provide a selective advantage against malaria infections. These observations suggest that as we continue to query the human genome for associations with blood cell phenotypes, integrating evidence of natural selection would be a powerful approach.

## 3. Genetic Modifiers of Disease Severity

Several human diseases, which afflict a large fraction of the human population, are characterized by abnormally low or high counts of the three main blood cell types, or some unusual values for their features or contents. Anemia is a decrease of RBC count and hemoglobin levels (<11 g/dL in women or <13 g/dL in men) and is characterized by a wide spectrum of symptoms from simple fatigue to heart failure [51]. The World Health Organization estimates that anemia affects 1.62 billion people in the World [52]. The main causes of anemia are poor nutrition and iron deficiency, infections (e.g., malaria) and RBC diseases such as the hemoglobinopathies. Although the effect size of an individual SNP associated with RBC count or hemoglobin levels is not sufficient to cause anemia, a combination of hemoglobin-reducing alleles at many SNPs could have an impact on the risk to develop this disorder. Maybe more importantly, without causing anemia itself, this genetic score could influence clinical severity in at-risk populations (e.g., children with a small number of hemoglobin-increasing alleles that live in a region where malaria is endemic). Since anemia is mostly frequent in Africa and South-East Asia, it is critical to continue to search for genetic associations with hemoglobin levels in these populations [52].

There are many other human diseases that are diagnosed, like anemia, through abnormal counts of the main blood cell types (e.g., cancers). One example is myeloproliferative neoplasms (MPNs), diseases of the bone marrow characterized by excess cell production [53]. By far the main cause of MPNs is a somatic gain-of-function mutation in the kinase gene *JAK2* (Val617Phe), which activates cell proliferation in the myeloid lineage [54,55], and changes platelet formation and reactivity [56]. It has never been tested whether SNPs associated with blood cell counts could modify complication risk in MPN patients with a *JAK2* (Val617Phe) mutation. For instance, MPN patients are at high risk of stroke, but it is unknown if such patients that also carry a large number of platelet-increasing alleles are at even higher stroke risk. Such analyses, on MPNs but also all other diseases characterized by a blood phenotype, are simple and could test the role that SNPs associated with normal variation in hematological traits may have on our risk to develop more severe disorders and related complications [18].

BCL11A Modifies Clinical Severity in Hemoglobinopathies

In adults, hemoglobin (HbA) is composed of two α- and two β-globin subunits that form a tetramer with the heme moiety to transport oxygen from the lungs to the different organs. Prior to birth, the *β-globin* gene is silent and the β-globin subunits are encoded by the *γ-globin* genes to form fetal hemoglobin (HbF). The switch from HbF to HbA production is a transcriptionally and epigenetically tightly regulated process [57]. For most healthy individuals, the switch itself has no clinical impact. However, for β-thalassemia and sickle cell disease patients with mutations in the *β-globin* gene, understanding and modulating the globin switch is currently the most promising therapeutic strategy. Conceptually, this is easy to appreciate: if the disease-causing mutations are in the *β-globin* gene, then re-activating *γ-globin* gene expression to form “normal” β-globin subunits would bypass the problem. This approach is supported by an extensive literature on the natural history of hemoglobinopathies and epidemiological studies [58]. For instance, it has been shown that sickle cell disease patients that normally produce more HbF have better survival prognostic and less severe disease complications than patients with low HbF levels [59,60,61].

Although as adults we mostly produce HbA, we continue to make residual levels of HbF. Inter-individual variation in HbF levels is highly heritable (*h*^2^ ~ 0.6–0.9) [2,62]. Genetic investigations, including GWAS, have identified common genetic variation at three loci (*BCL11A*, *HBS1L-MYB* and *β-globin*) that have strong phenotypic effects and that together explain almost half of the heritable variation in HbF levels [63,64,65,66]. These HbF-associated SNPs are also associated with clinical severity in β-hemoglobinopathy patients: transfusion-dependency in β-thalassemia and painful crises in sickle cell disease [65,67,68]. This again emphasizes the importance of HbF as a strong modifier of severity for these diseases.

*BCL11A* encodes a transcription factor that had no known function in the globin switch before its discovery in two GWAS for HbF levels [63,65]. Since then, we have learned that BCL11A is a potent transcriptional repressor of *γ-globin* gene expression and that its inactivation in the erythroid lineage can treat a sickle cell disease mouse model through re-activation of HbF production [69,70]. More recently, both genetic and molecular fine-mapping work has determined that HbF-associated SNPs located in a *BCL11A* intron disrupt en erythroid enhancer that controls *BCL11A* expression [71]. This model was confirmed by targeted deletion of the enhancer through genome engineering that blocked *BCL11A* expression and re-activated *γ-globin* gene expression and HbF production [16]. As genome editing methods are rapidly improving, this proof-of-concept experiment suggests a new therapeutic strategy in which the *BCL11A* enhancer would be deleted *ex vivo* in a hemoglobinopathy patient’s cells to re-activate HbF production, and the cells would then be transplanted back to the patient [72]. The characterization of *BCL11A* and its role in HbF production serves as a powerful example to illustrate the success of GWAS from new biology to potentially innovative therapy.

## 4. Orphan Blood Cell Diseases

Although we did not assess the statistical significance of the enrichment, we observed that many of the SNPs associated with blood cell traits are located near genes that are mutated in severe hematological disorders and inherited in a Mendelian fashion. These include SNPs near *HK1* (hemolytic anemia), *TMPRSS6*, *HFE* and *TFR2* (iron deficiency) or *TUBB1* (thrombocytopenia). This observation is similar to the situation of many other complex human phenotypes (e.g., lipids, height, diabetes) where GWAS have identified hypomorphic alleles near human syndrome genes for related phenotypes. As such, the long list of loci found by GWAS provides a framework to investigate human syndromes characterized by aberrant blood features, mapped to a chromosome arm by linkage studies, but where the gene culprit has not been identified yet.

To investigate this hypothesis, we queried the Online Mendelian Inheritance in Man (OMIM) database [73]. In a non-exhaustive search, we identified four such orphan diseases where the genomic locations overlap with SNPs identified by GWAS (Table 3). For three of the diseases, GWAS findings suggest a strong candidate gene (*IL5*, *LIPC*, *NUDT19*) for re-sequencing in affected individuals. As we continue to map these rare blood disorders, cross-referencing with GWAS hits may provide a strong filter to prioritize genes for genetic testing.

**Table 3:** Orphan human syndromes mapped to a chromosomal band and characterized by a blood cell phenotype. Only such syndromes that overlap with a locus identified by GWAS for the corresponding blood cell trait are included in this table. We generated this list by querying the Online Mendelian Inheritance in Man (OMIM) database with the following keywords: anemia, blood, hemoglobin, leukopenia, neutropenia, platelet, thrombocytopenia.

Mendelian genetics: orphan syndromes				Genome-wide association studies				
Locus	Disease	OMIM#	Description	SNP	Position	Phenotype	Candidate-gene(s)	Ref.
5q31	Familial eosinophilia	131400	Characterized by peripheral hypereosinophilia with or without other organ involvement	rs4143832	chr5: 131,862,977	Eosinophil count	*IL5*	[33]
6p21	Macroblobulinemia, susceptibility to Waldenstrom	153600	Malignant B-cell neoplasm characterized by lymphoplasmacytic infiltration of the bone marrow and hypersecretion of monoclonal immunoglobulin M (IgM) protein	rs2517524	chr6: 31,025,713	White blood cell	*HLA* region	[45]
15q21	Dyserythropoietic anemia, congenital type III	105600	Characterized by nonprogressive mild to moderate hemolytic anemia, macrocytosis in the peripheral blood, and giant multinucleated erythroblasts in the bone marrow	rs1532085	chr15: 58,683,366	Hemoglobin	*LIPC*	[18]
19q13	Transient erythroblastopenia of childhood	227050	Red blood cell aplasia	rs3892630	chr19: 33,181484	Mean corpuscular volume	*NUDT19*	[18]

## 5. Conclusions

GWAS have identified hundreds of loci that carry common genetic variants associated with RBC, WBC and platelet phenotypes. Many of these genetic associations still need to be linked to causal genes and genetic variants, yet because tractable cellular and animal models are available, this might be simpler for blood cell traits than it is for most complex human phenotypes. By design, GWAS interrogate common DNA variants, leaving untested low-frequency and rare sequence variation. The development of next-generation DNA sequencing platforms and exome genotyping arrays now provides the tools to test the role of this rarer genetic variation on blood cell phenotypes. Much criticism has been raised against GWAS because identified SNPs have poor predictive value; this is also true for SNPs associated with blood cell traits. However, this observation needs to be counter-balanced by the potential gain in improving our understanding of human biology in health and disease. GWAS blood cell trait loci provide new opportunities to study hematopoiesis, natural selection and the various ways common segregating DNA sequence variants can modify disease severity, paving the way for the development of more specific therapies.
